# National drug policy reform for noncommunicable diseases in low-resource countries: an example from Bangladesh

**DOI:** 10.2471/BLT.15.161117

**Published:** 2016-04-05

**Authors:** Sheikh Mohammed Shariful Islam, Md Tauhidul Islam, Anwar Islam, Anthony Rodgers, Clara K Chow, Aliya Naheed

**Affiliations:** a Non-Communicable Disease Initiative, International Center for Diarrhoeal Diseases Research, Bangladesh, 68 Shaheed Tajuddin Ahmed Sarani, Mohakhali, Dhaka-1212, Bangladesh.; b School of Health Policy & Management, York University, Toronto, Canada.; c Cardiovascular Division, The George Institute for Global Health, Sydney, Australia.; Correspondence to Sheikh Mohammed Shariful Islam (Email: shariful.islam@icddrb.org)

In recent years, the incidence of cardiovascular diseases, diabetes, cancers and chronic respiratory diseases has increased in epidemic proportions in many low-resource countries, including Bangladesh.^1^ Noncommunicable diseases accounted for an estimated 38 of 56 million deaths worldwide in 2012. Yet most of the deaths (74%, 28 million) occurred in low- and middle-income countries, where access to essential medicines to prevent and treat these diseases remains low.^2^^,^^3^ The Global Burden of Disease study projected that noncommunicable diseases will be the leading global cause of disability by 2030.^4^

Lack of access to essential medicines for noncommunicable diseases is a major challenge for the health systems in many low-resource countries, potentially contributing to increased mortality and morbidity in these countries.^5^ The World Health Organization (WHO) has recommended that every country should have a national drug policy that ensures access, quality and rational use of medicines as an integral part of its broader strategic health-care policy.^6^ It has also recommended that noncommunicable diseases be incorporated into the strategic plans of national drug policies.^7^ The WHO *Model list of essential medicines* contains 95 basic medicines for most noncommunicable diseases, including 16 new medicines for cancer that were added in May 2015.^8^ Despite the wide adoption of national drug policies in low- and middle-income countries, the majority of these do not include essential medicines for noncommunicable diseases.

Bangladesh is one of the pioneer countries to develop a national drug policy, launching its first in 1982. Based on the WHO essential medicines concept, the national drug policy identified 150 essential drugs for controlled pricing. The policy was instrumental in improving the supply and accessibility of quality essential drugs at an affordable price.^9^ However, during the 1980s and 1990s in Bangladesh the quality assurance of medicines started to be neglected, the price of local medicines increased and there was a shift in production from essential medicines to more profitable unregulated medicines by the major pharmaceutical industries. Despite being one of the world’s poorest countries, Bangladesh has made great progress over the last few decades in reducing maternal and child mortality, decreasing the burden of communicable diseases and improving life expectancy.^10^

In 2011, the Government of Bangladesh adopted a new national health policy, incorporating a strategic plan for the surveillance and prevention of noncommunicable diseases, along with a plan for the provision of essential drugs for these diseases.^10^ However, these policy documents did not mention specific medicines for noncommunicable diseases or requirements for their availability, price, quality assurance or rational use. 

Many issues prevented the optimal implementation of the essential drugs and noncommunicable diseases’ policies in Bangladesh: lack of specific financial resources; lack of leadership and accountability; insufficient planning and forecasting; inefficient procurement; coordination and monitoring; and other competing priorities. However, several countries have undertaken initiatives to promote access to medicines for these diseases. For example, the Brazilian government, through its popular pharmacy programme, partnered with private pharmacies to market subsidized medicines for noncommunicable diseases, a scheme that can be replicated in other countries.^11^

Most noncommunicable diseases can be treated or managed with a small range of low-cost generic medicines such as anti-hypertensives, statins and other cardiovascular drugs, analgesics, anti-asthmatics and some common anti-cancer drugs.^2^ However, many of the essential medicines for diabetes in Bangladesh are too expensive for the general population or are not available in government facilities.^12^ A study in 2016 found that for patients with diabetes the cost of drugs accounted for the largest share of their total health-care expenditure.^13^ From its inception the Bangladesh national drug policy failed to include some of the essential medications from the WHO essential list for cancer, diabetes, cardiovascular diseases, respiratory diseases and mental illnesses. The 209 medicines in the Bangladesh essential medicines list for 2008 included only 30 drugs for noncommunicable diseases (Table 1). Furthermore, many medicines for noncommunicable diseases are not widely prescribed or available, as pharmaceutical companies and physicians might prefer to promote more expensive, profit-yielding drugs. Counterfeit versions of costly drugs may also be available in the market and might be promoted by local pharmacies for higher profits. Moreover, several pharmaceutical companies in Bangladesh lack heating, ventilation, air-conditioning systems and warehouse facilities that conform to internationally mandated standards.^15^ In addition, the Bangladesh drug regulatory authority has constrained resources to monitor the quality of medicines produced and imported into the country.

**Table 1:** Number of medicines for noncommunicable diseases in the Bangladesh and World Health Organization lists of essential drugs

Category	WHO list		Bangladesh list	
	No. of medicines		No. of medicines	Names
**Noncommunicable diseases**				
Cancers	40		7	Cisplatin, cyclophosphamide, methotrexate, procarbazine, tamoxifen, vinblastine, vincristine
Diabetes	5		4	Glibenclamide, gliclazide, insulin, metformin
Cardiovascular diseases	28		13	Atenolol, digoxin, enalapril, furosemide, glyceryl trinitrate, isosorbide dinitrate, nifedipine, methyldopa, procainamide, propranolol, spironolactone, verapamil, warfarin
Mental health disorders	17		5	Amitryptiline, fluorouracil, fluphenazine, haloperidol, lithium carbonate
Respiratory diseases	5		1	Salbutamol
**All diseases**	410		209	N/A

N/A: not applicable; WHO: World Health Organization.

Sources: WHO *Model list of essential medicines*, 2015^8^ and *Bangladesh List of essential drugs*, 2008.^14^

In 2012, WHO Member States agreed on a set of indicators for drugs and technologies for noncommunicable diseases. These included assessing the availability of 80% of generic essential medicines for noncommunicable diseases in public and private facilities, and a voluntary target that 50% of the population at risk of heart attacks and stroke receive appropriate drug therapy and counselling.^2^ The Noncommunicable Diseases Alliance recommends the critical evaluation of guidelines, prices, availability, accessibility, financing and quality of medicines and services for noncommunicable diseases; that pricing policies be reviewed ; and the capacity of the national drug regulatory authority be strengthened to ensure the quality, safety and efficacy of essential medicines and technologies in the country.^5^ These recommendations place additional pressure on low- and middle-income countries to improve access to, and availability of, medicines for noncommunicable diseases. However, care for such diseases is still inadequate within the primary-care setting in Bangladesh and other low-resource countries, primarily due to a shortage of equipment, medicines and trained staff, and a focus on maternal and child health. This creates a considerable challenge to incorporate all the WHO essential medicines for noncommunicable diseases into the national essential medicines list.

In most low-resource countries, multiple barriers exist to reforming the national drug policy, which are often complex and go beyond simple financial constraints. These include political instability, bureaucratic inertia, resistance from entrenched interest groups eager to protect the existing policy, government financial constraints, and policy issues that often transcend borders.^6^ In many cases, the lack of national clinical guidelines for noncommunicable diseases hinders the selection of essential medicines for these diseases. Generally, the inclusion of a drug in the national drug policy mandates its provision in government-funded care settings. This is made more complex by the high cost of medicines for cancer, and some patent drugs with little or no clinical benefits over existing standard medicines, which resource-poor governments cannot afford to provide. In many low- and middle-income countries decisions on the cost‒effectiveness of drugs therefore have to be made, which in themselves pose considerable technical and resource challenges for already stretched departments of health. Other potential barriers to national drug policy reform may include weak supply systems from storage facilities to clinics; weak regulations and quality assurance; limited capacity of national drug regulatory authorities; irrational drug prescribing and dispensing; and limited market competition for critical essential medicines. In addition, there may be opposition to a national drug policy from clinicians, due to fear of loss of clinical freedom, and from pharmacies and pharmaceutical companies, due to competing interests.

Evidence from several countries suggests that a comprehensive approach is required to overcome these barriers. In Sweden, the drug and therapeutics committee in Stockholm created a regional list of 200 essential drug recommendations for use in ambulatory care, and demonstrated 87% adherence to the recommendations by physicians.^16^ Identifying and implementing strategies to improve the use of essential medicines is needed using strong legislative frameworks, and political will, commitment and support for national drug policies. The national drug policy needs to be embedded in the national health policies and health systems. Along with strengthening the national drug regulatory authorities, other essential elements for a sustainable national drug policy include greater allocation of financial resources, and identifying alternative mechanisms such as pre-payment, tax, pricing policies, promoting generic medicines and negotiations with global trade agreements (e.g. measures compliant with the Trade-Related Aspects of Intellectual Property Rights). Using central procurement and distribution can increase the availability of high-quality essential medicines. Other measures include promoting rational use of medicines, preventing inappropriate drug promotion, capacity-building for human resources, promoting drug information centres, publishing drug bulletins, providing consumer information and education, and strengthening multisectoral collaboration.^17^

Noncommunicable diseases impose a heavy financial burden on affected individuals, families and the economy in countries such as Bangladesh. The responsiveness of the Bangladesh national drug policy to the country’s rapid epidemiological transition and increased burden of noncommunicable diseases remains weak. Ensuring accessibility, rational prescribing and quality of essential medicines for these diseases needs to be prioritized, alongside strategies such as lifestyle changes and tobacco and other taxes. In short, there is an urgent need to reform the national drug policy to incorporate noncommunicable diseases within its mandate, so that treatment and essential drugs for these diseases could be made available and accessible to all, especially poor and disadvantaged people. Potential approaches that Bangladesh and other low-resource countries could apply in the short, medium and long term include: (i) establishing national treatment guidelines and protocols for noncommunicable diseases; (ii) increasing the availability and affordability of medicines for noncommunicable diseases through central procurement and distribution; (iii) increasing government subsidies for noncommunicable diseases’ medicines through tax revenue, for example by imposing taxes on sugary drinks, or increasing tobacco and alcohol taxes; and (iv) developing sustainable mechanisms for promoting accessibility to noncommunicable diseases’ medicines for all patients, for example via sale of government-subsidized medicines in community pharmacies.

Such a reform of the national drug policy would not only strengthen the health system and help achieve universal health coverage, but also reduce the mortality and morbidity attributable to noncommunicable diseases in these countries.
